# Do surrogates predict patient preferences more accurately after a physician-led discussion about advance directives? A randomized controlled trial

**DOI:** 10.1186/s12904-022-01013-3

**Published:** 2022-07-12

**Authors:** Catarina Sampaio Martins, Iva Sousa, Cláudia Barros, Alexandra Pires, Luisa Castro, Cristina da Costa Santos, Rui Nunes

**Affiliations:** 1 Palliative Medicine Service of Centro Hospitalar de Tràs-Os-Montes E Alto Douro, 5000-508 Vila Real, Portugal; 2 MEDCIDS‐Department of Community Medicine, Information and Decision in Health, University of Porto, 4200‐450 Porto, Portugal; 3 CINTESIS‐Center for Health Technology and Services Research, 4200‐450 Porto, Portugal; 4 School of Health of the Polytechnic of Porto, 4200‐450 Porto, Portugal; 5 Department of Community Medicine, Information and Health Decision Sciences, Faculty of Medicine, MEDCIDS, University of Porto, Rua Dr. Plácido da Costa, 4200-319 Porto, Portugal; 6 Faculty of Medicine, CINTESIS, Centre for Health Technology and Services Research, University of Porto, Porto, Portugal; 7 Faculty of Medicine, MEDCIDS‐Department of Community Medicine, Information and Decision in Health, University of Porto, 4200‐450 Porto, Portugal

**Keywords:** Palliative Care, Advance Directives, Caregivers, Decision-making

## Abstract

**Background:**

Caregivers frequently assume the role of surrogate decision-makers but often are unable to accurately predict patients’ preferences. This trial aims to find if the use of the Advance Directives documents as a communication tool, improves the agreement between patients and caregivers.

**Methods:**

This trial occurred in a palliative care service of a Portuguese hospital center. A prospective, single-blinded, controlled, randomized trial, enrolling patients and caregivers as a dyad was conducted. Participants individually fulfilled an Advance Directive document, in which patients reported their end-of-life preferences and caregivers reported their decisions as patients’ health surrogates. Dyads were randomly assigned to the Intervention or the Control group, in which the physician respectively promoted an open discussion about patients’ Advance Directives or evaluated patients’ clinical condition. Caregivers’ Advance Directives as surrogates were collected one month later. Proportions of agreement and Cohen’s κ were used to access agreement and reliability, respectively, between the dyads.

**Results:**

Results from 58 dyads were analyzed. We observed an improvement in agreement between the caregivers’ answers and the patients’ wishes on two-thirds (8/12) of the answers, in the Intervention group, contrasting to one-quarter (3/12) of the answers, in the Control group, despite statistical significance in differences wasn´t obtained.

**Conclusions:**

Although not reaching statistical significance, the results suggest that discussions of advance directives with physicians may lead to better prepared surrogates.

**Trial registration:**

ClinicalTrials.gov ID NCT05090072. Retrospectively registered on 22/10/2021.

## Background

In palliative care, great concern is given to the comfort and dignity that each patient experiences while the disease progresses. Therefore, health care practitioners must encourage the patients’ participation in decision-making and understand their wishes and preferences. However, in some circumstances, patients become incapable of reporting their preferences, delegating their health care decisions to their caregivers. Consequently, their loved ones and physicians must delineate all the strategies and goals regarding their end-of-life treatments.

Caregivers are requested to decide on behalf of the patients and assume the role of surrogate decision-makers [1]. However, in a systematic literature review, Shalowitz (2006) [2] found that the patients’ surrogates are unable to accurately predict their preferences in about one-third of the cases [2]. Particularly in the hospice context, Engelberg (2005) [3], who analyzed 92 pairs of patient/surrogates, found discrepancies regarding the patients’ preferences in the last week of their lives, and their surrogates’ understanding of those preferences, with a median number of concordant items between patients and surrogates of 14 in 30. These authors concluded that families predicts only about half of the end-of-life experiences that patients consider important [3]. Moreover, caregivers might not have the motive to decide according to the patients’ wishes but instead, assume the strategy they consider more appropriate to their own end-of-life period [4].

Across the world, Advance Directives emerge as an essential document to promote patients’ autonomy until the end of their lives, ensuring that their wishes are fulfilled and respected [5]. However, in Portugal, its use is scarce, and its existence is unknown of in many places. In Portugal, on the first week of 2020, official records stated that only 29,347 citizens had their Advance Directives registered, representing 0.00286% of the global population.

The Portuguese Advance Directives [6] reflect the individuals’ last wishes and enable the designation of a health surrogate. This chosen person is frequently a close family member who assumes the responsibility of making decisions on behalf of his/her relative. According to Emanuel (2008) [7], these two modalities of expressing people’s end-of-life preferences, whether by writing an Advance Directive document or by nominating a surrogate decision-maker, are complementary, as the surrogates’ accuracy is incomplete without their relatives’ orientation [7]. Considering previous literature (Wittich, 2013) [1], we must assume that when patients write down their Advance Directives, the chosen surrogates may be unable to predict their last wishes if they do not discuss and clarify them beforehand [1].

We aim to determine if the physicians’ use of the Advance Directives [6] document in palliative care, as a communication tool between patients and their nominated surrogates, improves the agreement and reliability between patients and surrogates in their decisions. Changes in the agreement between the caregivers’ answers as surrogates, and the patients’ decisions for their own end-of-life care, before and after the physicians’ intervention will be the primary outcome of interest.

## Methods

### Study design, aim and setting

This trial was a prospective, single-blinded, controlled, and randomized study analyzing whether the Advance Directives’ [6] discussion between patients and caregivers, promoted by a palliative care physician, improves concordance between them concerning the patients’ last wishes, therefore improving the surrogates’ accuracy when they decide and act on behalf of the patients.

This trial was conducted in the Trás-os-Montes and Alto Douro Hospital Center, a central hospital in Portugal’s North inland, that served an estimated population of 465.000 habitants. We recruited patients referred to all three units of the palliative care service from September 2018 to September 2019.This trial followed all the ethical procedures under the Declaration of Helsinki, and the Ethics Committee of Trás-os-Montes and Alto Douro Hospital Center approved it on 18 June 2018 (Doc nº 245/2018).

Patients and caregivers were assigned to the study in the palliative care service, in their first consultation. An experienced nurse performed the patients and caregivers’ assessments for eligibility. The patients who were alone, screened as eligible to the study had another appointment scheduled with their caregiver, to determine the eligibility of the pair as a dyad patient/surrogate. After an exhaustive explanation of its content, all the participants gave written consent to enroll in the trial. Furthermore, we assured the possibility of dropping out at any moment during the study, to all the participants that expressed this desire.

### Participants

The patients’ inclusion criteria were adults aged 18 and over, referred to the palliative care service with a chronic, progressive, and incurable disease, ability to comprehend, write and speak the Portuguese language, and with the absence of major cognitive disorders (Portuguese validated Mini Mental State Test [8, 9] score superior to 22, 25 or 27, according to the patients’ education level). Also, the patients’ acceptance to participate in the trial and their ability to nominate a caregiver as their surrogate in decision-making had to be guaranteed. The caregivers’ inclusion criteria were adults aged 18 and over, being nominated by patients as their surrogate decision-maker, with the ability to comprehend, write and speak the Portuguese language, and their acceptance to participate in the trial.

### Interventions

This trial occurred in two phases, within a month’s interval. At baseline (phase 1), before any intervention, data were collected on the patients’ and caregivers’ sociodemographic features. The dyad was then separated, and each element alone filled the Advance Directives’ document [6] considering the patients’ preferences for end-of-life care. Caregivers were asked to answer the document as the patients’ surrogates and decide on their behalf. No communication between the pair was allowed before the completion of the formulary. Dyads were then randomly assigned to two groups—Intervention and Control groups.

In the Intervention group, the palliative care physician engaged patients and their caregivers in an open discussion, considering the patients’ answers to the Advance Directives [6] and exploring and clarifying all doubts and questions prompted by the document. In the Control group, the palliative care physician underwent a conference with both patients and caregivers and evaluated the patients’ symptoms through the Edmonton Symptom Assessment System [10], adapted to the Portuguese population. In the Control group intervention, the physician did not approach the Advance Directives’ [6] theme. Both interventions occurred in the clinical rooms of the palliative care service, on the scheduled day with both patients and caregivers, with a time range of 40 to 50 min. At phase 2, one month after the first interview, caregivers were asked to fill out another Advance Directives’ [6] formulary as the patients’ surrogates.

### Outcomes

In both randomized groups, the primary goal was to analyze the improvement in agreement between the patients and caregivers’ answers in the Advance Directives document, after the palliative care physician’s intervention. The document’s Sect. 1 comprises three possible clinical scenarios, and Sect. 2 asks 12 yes/no questions as relevant to the scenarios, which yields 36 answers for each participant.

Agreement between patients and caregivers’ answers, in both Intervention and Control groups, in phase 1 and phase 2, were analyzed. Themes with significant concordance were found, and the statistical significance of the results was calculated.

### Instruments

The Portuguese official Advance Directives [6] was the central instrument used in this trial, consisting of a two-step approach document. Table 1 lists the Portuguese Advance Directives’ [6] content.

**Table 1:** Portuguese Advance Directives Content [6]: the document presents two sequential sections to answer. In Sect. 1, participants choose the clinical scenarios they intend to apply the answers of Sect. 2 questions, up to 3 different scenarios which refer to medical conditions that render them unable to express their wishes or exert their autonomy

Scenario 1	Diagnosis of incurable and terminal disease
Scenario 2	No expected recovery, according to state of art
Scenario 3	Unconsciousness with irreversible neurologic or psychiatric disease complicated by respiratory, renal, or cardiac disfunction
Question 1	Do not receive cardiopulmonary resuscitation
Question 2	Do not be submitted to invasive vital organ support
Question 3	Do not receive artificial nutrition and hydration just to slow up the natural death process
Question 4	To participate in experimental studies or investigation trials
Question 5	Do not be submitted to experimental treatments
Question 6	Do not be submitted to experimental studies or investigation trials
Question 7	To interrupt previously consented experimental treatments or investigation trials participation
Question 8	Do not authorize blood and derivates transfusions
Question 9	To receive palliative care and minimal oral or subcutaneous hydration
Question 10	To be administered effective and necessary pain killers and other symptom control drugs
Question 11	To receive spiritual assistance when invasive life support is ended
Question 12	Be accompanied by the following person _____________________ when invasive life support is ended

As shown in Table 1, this document presents two sequential sections to answer. In Sect. 1, the participants choose the clinical scenarios they intend to apply the answers of Sect. 2, up to 3 different scenarios. These scenarios refer to medical conditions that render them unable to express their wishes or exert their autonomy. Specifically, Sect. 1 describes situations of “incurable terminal disease”, “irreversible comatose state, caused by neurological or psychiatric disease, aggravated by respiratory, renal or cardiac intercurrence” or “situation with no recovery perspective, according to the state-of-the-art”. Then, in Sect. 2, participants express their wishes, answering the 12 sentences regarding end-of-life preferences, like “cardio-respiratory resuscitation”, “invasive organ support”, “artificial tube feeding and hydration”, “blood transfusions”, “spiritual help”, among others.

We registered each answer as Yes or No, according to the participants' decisions. Since all the participants answered the 12 items in Sect. 2 in the same way for all the 3 scenarios in Sect. 1, the analysis was applied to the 12 items’ responses.

### Sample

The target population consisted of the group of patients referred to the palliative care service of the Trás-os-Montes and Alto Douro Hospital Center. We approached the patients sequentially according to their consultation date to find eligibility to the trial and the fulfillment of the inclusion criteria.

### Randomization and blinding

Simple individual randomization was previously achieved (www.random.org) [11]. The randomization sequence was disclosed through a sealed envelope only after the dyads’ enrollment on trial, after all the sociodemographic data were collected, and all the Advance Directives’ documents were filled, to ensure the investigator’s proper concealment.

Both patients and caregivers were blinded to the assigned group during the trial. In both groups (Intervention and Control), the dyads were engaged in a conference meeting, in the same room, with the same average duration, and with the same investigator.

### Statistical analysis

Categorical variables were described by absolute and relative frequencies. Age was described by the mean and standard deviation (mean ± SD), as its distribution did not deviate from normality, according to visual analysis of histograms and confirmed with the Shapiro–Wilk’s test of normality. The sociodemographic characteristics were compared between patients and caregivers with Chi-Square and independent sample t test.

The terms ‘‘reliability’’ and ‘‘agreement’’ are often used interchangeably. However, the two concepts are conceptually distinct [12]. Reliability may be defined as the ratio of variability between subjects or objects to the total variability of all measurements in the sample. Therefore, reliability can be defined as the ability of a measurement to differentiate between subjects or objects. On the other hand, agreement is the degree to which scores, or ratings are identical. Both concepts are important because they provide information about the quality of measurements. Consequently, as recommended by the Guidelines for Reporting Reliability and Agreement Studies (GRRAS) [12] the reliability was assessed with Cohen’s kappa and agreement with Proportions of Agreement.

Agreement between patients and their caregivers was assessed (in each question) with the Proportions of Agreement (PA), with respective 95.0% confidence intervals. According to Grant, if the lower limit of the 95.0% Confidence Intervals for Proportions of Agreement was lower than 0.50, agreement was considered to be poor [13]. Maximal Proportion of Agreement in each question (PA = 1), was reached when all the patients’ and caregivers’ dyads fully agreed on all answers. Proportions of Agreement was also compared between Control and Intervention groups.

Reliability was accessed with Cohen’s kappa (κ) [14]. We assumed Landis and Koch’s (1977) [15] interpretation of the κ value. According to these authors, a κ value of 0.20 represents slight reliability, a value between 0.21 and 0.40 fair reliability, a value between 0.41 and 0.60 moderate reliability, a value between 0.61 and 0.80 substantial reliability, and a value of 0.81–1.00 indicates almost perfect reliability [15].

Descriptive data analysis was performed using SPSS® Statistics (version 26.0; SPSS Inc., Chicago, IL, USA). Proportions of Agreement and Cohen’s κ with respective confidence intervals were computed using packages “obs. agree” (Henriques, 2013) [16] and “psych” (Revelle, 2020) [17] from R software, v 3.4.0 (R Core Team, 2020) [18].

We used a significance level of 0.05 in all the statistical analyses.

## Results

**Fig. 1:**
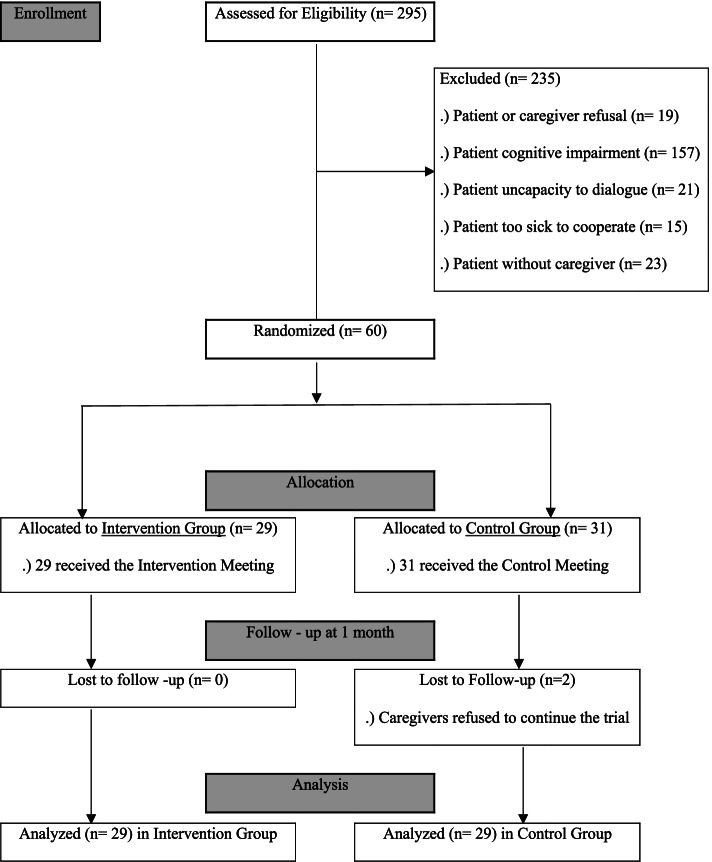
Consort 2010 Flow Diagram

From September 2018 to September 2019, 295 patients were assigned to the trial, but only 60 dyads met all the inclusion criteria and were enrolled. After randomization, 29 dyads were allocated to the Intervention group and 31 dyads to the Control group. Two caregivers in the Control group decided to drop out, as patients suffered clinical deterioration, resulting in 29 dyads in both groups to be analyzed (Fig. 1). The Intervention group registered no losses to follow-up.

In Table 2, we can observe the sociodemographic data of both patients and caregivers, according to their allocation group (Intervention group, *n* = 29, and Control group, *n* = 29). As shown, data regarding the patients’ age and gender were considerably similar in both Control and Intervention groups, with no significant differences. Caregivers also had no significant differences in gender proportions in both groups; however, we noticed significant differences regarding their age distribution as caregivers were significantly younger in the Control group (average ± SD age was 62.5 ± 13.14 years in the Intervention group versus 54.5 ± 13.27 years in the Control group). Data distribution from marital status, education level, and provenience were also similar and did not differ significantly, for patients and caregivers, in both Intervention and Control groups. The patients’ clinical performance was assessed with the Palliative Performance Scale (PPS) [19], and no significant differences were noticed between the two groups of patients, in these scores’ distribution (Table 2). Caregivers’ clinical performance has not been evaluated.

**Table 2:** Description of Patients and Caregivers Sociodemographic and Patients’ clinical performance assessed with the Palliative Performance Scale (PPS) and comparison between the Intervention and Control groups both for patients and caregivers

	**Intervention Group (n Dyads = 29)**		**Control Group (n Dyads = 29)**		***p*** **-value**	***p*** **-value**
	Patients	Caregivers	Patients	Caregivers	Patients	Caregivers
**Gender n (%)**					0.599	0.557
Female	13 (44.8)	22 (75.9)	15 (51.8)	20 (68.9)		
Male	16 (55.2)	7 (24.1)	14 (48.2)	9 (31.1)		
**Age (years, mean ± SD)**	70.9 ± 11.80	62.5 ± 13.14	69.7 ± 14.83	54.5 ± 13.27	0.726	0.025
**Marital status n (%)**					0.588	0.125
Single / Divorced / Widower	10 (34.5)	4 (13.8)	12 (41.4)	8 (28)		
Married or similar	19 (65.5)	25 (86.2)	17 (58.6)	21 (72)		
**Education level n (%)**					1.000	1.000
Iliterate / Primary School	22 (75.9)	10 (34.5)	22 (75.9)	10 (34.5)		
Medium/High school	7 (24.1)	14 (48.3)	7 (24.1)	14 (48.3)		
University	0 (0.0)	5 (17.2)	0 (0.0)	5 (17.2)		
**PPS Score n (%)**					0.721	NA
Score < 50	3 (10.3)	NA	2 (6.9)	NA		
50 ≤ Score < 70	15 (51.7)	NA	18 (62.1)	NA		
70 ≤ Score < 90	10 (34.5)	NA	7 (24.1)	NA		
Score ≥ 90	1 (3.5)	NA	2 (6.9)	NA		

*NA* Not Applicable, *PPS* Palliative Performance Scale, *SD* Standard deviation

Table 3 shows the agreement between patients and their caregivers, assessed with the Proportion of Agreement (PA) for the 12 questions of the Portuguese Advance Directives [6], from the Intervention and Control group in both phases. Table 4 describes the reliability between patients and their caregivers, assessed with the kappa statistics for the same questions.

**Table 3:** Proportion of Agreement (PA) between Patients and Caregivers for the Intervention and the Control Group and difference between phase 1 and phase 2 with respective p value. Bold means not poor agreement

Questions	Phase 1		Phase 2		Intervention	Control
	**Intervention**	**Control**	**Intervention**	**Control**	**Difference**	**Difference**
	PA [95.0% CI]	PA [95.0% CI]	PA [95.0% CI]	PA [95.0% CI]	**Phase 2 – Phase 1** p-value	**Phase 2 – Phase 1** p-value
**Q1**	**0.72**	0.66	**0.83**	0.62	0.11	-0.04
	**[0.55;0.86]**	[0.48;0.83]	**[0.69;0.97]**	[0.41;0.79]	0.320	0.753
**Q2**	**0.72**	**0.72**	**0.72**	0.66	0.00	-0.06
	**[0.55;0.86]**	**[0.55;0.86]**	**0.55;0.86]**	[0.48;0.79]	1.000	0.624
**Q3**	0.59	0.62	**0.76**	0.66	0.17	0.04
	[0.41;0.76]	[0.41;0.79]	**[0.59;0.9]**	[0.48;0.79]	0.171	0.753
**Q4**	0.48	0.66	0.48	0.62	0.00	-0.04
	[0.28;0.66]	[0.48;0.83]	[0.28;0.62]	[0.45;0.79]	1.000	0.753
**Q5**	0.45	0.66	0.55	0.55	0.10	-0.11
	[0.28;0.62]	[0.45;0.83]	[0.34;0.72]	[0.38;0.72]	0.450	0.396
**Q6**	0.52	0.62	0.55	0.62	0.03	0.00
	[0.31;0.69]	[0.45;0.79]	[0.34;0.72]	[0.45;0.79]	0.824	1.000
**Q7**	0.52	0.62	0.59	0.55	0.07	-0.07
	[0.34;0.69]	[0.41;0.79]	[0.41;0.76]	[0.38;0.72]	0.595	0.592
**Q8**	**0.76**	0.59	**0.83**	0.41	0.07	-0.18
	**[0.62;0.93]**	[0.41;0.76]	**[0.69;0.97]**	[0.24;0.59]	0.523	0.172
**Q9**	**1.00**	**0.97**	**1.00**	**0.97**	0.00	0.00
	**[1.00;1.00]**	**[0.9;1.00]**	**[1.00;1.00]**	**[0.90;1.00]**	1.000	1.000
**Q10**	**1.00**	**0.97**	**1.00**	**0.97**	0.00	0.00
	**[1.00;1.00]**	**[0.9;1.00]**	**[1.00;1.00]**	**[0.90;1.00]**	1.000	1.000
**Q11**	**0.83**	**0.69**	**0.97**	**0.79**	0.14	0.10
	**[0.69;0.93]**	**[0.52;0.86]**	**[0.9;1.00]**	**[0.66;0.93]**	0.078	0.389
**Q12**	**0.76**	**0.86**	**0.90**	**0.90**	0.14	0.04
	**[0.59;0.9]**	**[0.72;0.97]**	**[0.79;1.00]**	**[0.76;1.00]**	0.159	0.642

**Table 4:** Reliability between Patients and Caregivers for both the Intervention and the Control Group

Questions	Phase 1		Phase 2	
	**Intervention**	**Control**	**Intervention**	**Control**
	Cohen’s κ [95.0% CI]	Cohen’s κ [95.0% CI]	Cohen’s κ [95.0% CI]	Cohen’s κ [95.0% CI]
**Q1**	0.37 fair	0.16 fair	0.61 **substantial** ^a^	0.19 fair
	[0.03;0.71]	[-0.2;0.52]	[0.32;0.91]	[-0.12;0.51]
**Q2**	0.31 -fair	0.27 fair	0.31—fair	0.26 fair
	[-0.07;0.69]	[-0.1;0.63]	[-0.07;0.69]	[-0.03;0.55]
**Q3**	0.15 slight	0.21 fair	0.50 **moderate** ^a^	0.29 fair
	[-0.22;0.51]	[-0.15;0.57]	[0.17;0.82]	[-0.06;0.64]
**Q4**	-0.17 slight	0.3 fair	0.05 slight	0.24 fair
	[-0.51;0.16]	[-0.05;0.65]	[-0.24;0.35]	[-0.11;0.59]
**Q5**	-0.21 slight	0.29 fair	0.18 slight	0.1 slight
	[-0.54;0.11]	[-0.06;0.64]	[-0.11;0.46]	[-0.26;0.46]
**Q6**	-0.12 slight	0.23 fair	0.17 slight	0.24 fair
	[-0.46;0.22]	[-0.13;0.58]	[-0.12;0.45]	[-0.11;0.59]
**Q7**	-0.12 slight	0.21 fair	0.21 **fair** ^a^	0.06 slight
	[-0.46;0.22]	[-0.15;0.57]	[-0.09;0.51]	[-0.3;0.43]
**Q8**	0.37 fair	0.10 slight	0.55 **moderate** ^a^	-0.22 slight
	[-0.01;0.75]	[-0.25;0.45]	[0.2;0.9]	[-0.57;0.12]
**Q9**	NA	0 slight	NA	0 slight
		[0;0]		[0;0]
**Q10**	NA	0 slight	NA	0 slight
		[0;0]		[0;0]
**Q11**	0.34 fair	-0.18 slight	0.87 **perfect** ^a^	0.28 **fair** ^a^
	[-0.11;0.8]	[-0.3;-0.06]	[0.62;1]	[-0.16;0.71]
**Q12**	-0.11 slight	0 slight	-0.05 slight	0.36 **fair** ^a^
	[-0.22;0.01]	[0;0]	[-0.12;0.02]	[-0.16;0.89]

^a^reliability increase in the second phase from one category to other (slight < fair < moderate < substantial < perfect),

Data from the Intervention group (*n* = 29) on Table 3 show poor agreement between patients and caregivers, in 5/12 of the answers at phase 1 (baseline) (Q3, Q4, Q5, Q6, Q7). These questions concerned themes like artificial tube feeding and hydration (Q3, PA = 0.59), participation in experimental studies or treatments (Q4, PA = 0.48, Q5, PA = 0.45), submission or interruption of previous consented experimental studies and treatments (Q6 and Q7, PA = 0.52). Also at baseline, a better agreement between the dyads of the Intervention Group was noticed in 4/12 answers on themes about cardiopulmonary resuscitation (Q1, PA = 0.72), invasive organ support (Q2, PA = 0.72), blood transfusions (Q8, PA = 0.76), and company at end-of-life (Q12, PA = 0.76). However, a greater baseline agreement between the dyads (PA > 0.8) was seen on only 3/12 questions, that reflected answers about palliative care measures (Q9, PA = 1), administration of symptomatic control drugs (Q10, PA = 1), and end-of-life spiritual assistance (Q11, PA = 0.83) (Table 3).

Reliability was not applicable in Q9 and Q10, as all patients and caregivers answered Yes. All other Questions have slight to fair reliability between the patients and caregivers’ answers at phase 1 (baseline), in the Intervention Group (Table 4).

Although difference of PA from phase 1 to phase 2 did not reach statistical significance, we observed that in phase 2, in the Intervention group, agreement and reliability estimates improved in most of the questions (Table 3 and Table 4). The caregivers’ answers concerning artificial tube feeding and hydration (Q3), which presented the most considerable agreement improvement with patients’ wishes, increased from 0.59 to 0.76 from phase 1 to phase 2 in the Intervention group. However, this improvement did not reach statistical significance (Table 3). Also, the reliability has increased from slight to moderate from phase 1 to phase 2 in the Intervention group considering Question 3 (Table 4).

In the Intervention group, major and subjective themes like cardiopulmonary resuscitation (Q1) and blood transfusions (Q8), also registered more concordant answers between patients and caregivers on phase 2, increasing from fair to substantial reliability and from fair to moderate reliability, respectively. Similarly, questions about spiritual assistance (Q11) and company at end-of-life (Q12) registered an increase in agreement, reaching an almost perfect agreement between patients and caregivers, in phase 2 of the Intervention group (PA = 0.97 and PA = 0.9, respectively). However, none of these differences between phase 1 and phase 2 was statistically significant.

Comparing these results with those from the Control group we can observe that similarly to the Intervention group, at baseline (phase 1) the reliability between patients and caregivers in the Control group was slight to fair in all questions (Table 4), and in 7 of the 12 questions (58.3%) the agreement was poor (Table 3). However, a better agreement between patient and caregiver was observed in answers regarding the same themes of palliative care measures, administration of therapeutical drugs, and company at the end-of-life.

Unlike the Intervention group, data from the Control group in phase 2, did not show notable improvement. In fact, only 25.0% of the answers (Q3, Q11, Q12) showed a Proportion of Agreement rise. However, when considering the Intervention’s group results, 8 answers (66.7%) showed an improvement in Proportion of Agreement and no answer evidenced less agreement in the phase 2 (Table 3). The remaining 9 answers in the Control group maintained or reduced the Proportion of Agreement score in phase 2, thus declining any potential benefit of the Control group intervention, on the agreement improvement between patient and caregiver. Even in some central themes, like cardiopulmonary resuscitation (Q1) we observed a Proportion of Agreement score reduction, in phase 2 of the Control group. Moreover, only Q11 and Q12 increased reliability from slight to fair in this group (Table 4).

## Discussion

Thiede (2020) [20], in a randomized controlled trial involving the engagement of patients and their surrogates on Advance Care Planning, reported that 72.7% of the surrogates felt very prepared to decide on behalf of the patients, although reporting other factors besides Advance Directives influencing surrogates’ sense of preparedness [20].

In this trial’s phase 1, we found poor concordance between the patients and caregivers’ answers to the Advance Directives’[6] documents, when caregivers were asked to act as patients’ surrogates, and decide their end-of-life care. These facts are worrying and serious, as the health care that patients receive might not represent their real wishes at the end of their lives.

The Intervention group results in phase 2 supports the intervention, using the Advance Directives model as a communication tool between patients and caregivers. The agreement estimates remained the same or improved in all questions (12/12), although not reaching statistical significance.

In the Control group, the improvement in agreement was low (3/12 questions). In half of the questions (6/12), agreement estimates even reduced, denoting the lack of knowledge that caregivers have about the patients’ last wishes and the inability of the Control group meeting to improve this knowledge.

Interestingly, the more concordant answers on phase 1, in both Intervention and Control groups (Q9 and Q10), concerned the administration of palliative care, hydration and symptom control drugs. We hypothesize that the maintenance of these good agreement estimates on phase 2, might reflect the caregivers’ consolidated knowledge about the patients’ wishes on these subjects, but as patients’ and caregivers’ answers were homogeneous and reliability was not applicable in the Intervention group, we must do this assumption cautiously.

These trial’s results also suggest that, even when patients and caregivers both register their Advance Directives, as they did in the Control group, this practice is not sufficient to promote their subsequent spontaneous discussion in family reunions or in more private dialogues. It seems that the physicians’ intervention is eventually needed to encourage this discussion, which potentially improves the caregivers’ ability to act as more competent health surrogates.

A recent review by Su (2019) [21] highlights caregivers’ unpreparedness to act as the patients’ surrogates and emphasizes the importance of health professionals’ support in making end-of-life decisions. Final recommendations focus on good communication habits between health professionals, surrogates, and patients, not only to guarantee that the patients’ preferences are respected but also to mitigate the caregivers’ burden of the decision-making process [21].

We noticed several limitations in this trial. Nearly 80.0% (*n* = 235) of the patients assessed for eligibility were excluded due to several conditions (Fig. 1), the main reason being patients’ cognitive impairment (*n* = 157), reflected by a Mini-Mental state test [8, 9] score below the predetermined limit. These data mirror the rural characteristics of the studied population, with relatively low education levels. Consequently, we find it possible that the restriction of the inclusion criteria to patients with a good cognitive capacity might have created some selection bias. Still, we assumed that the characteristics of this trial demanded good mental ability, so participants could understand the delineated goals. Other exclusion conditions were patient or caregiver refusal (*n* = 19) and patients being too sick to cooperate (*n* = 15). The late referral to palliative services might be contributing to these numbers. Frequently, patients present to a first palliative care consultation with advanced oncologic conditions and clinical deterioration, contributing to a low predisposition and capacity to engage in scientific trials.

We could notice mild differences in the patients’ clinical performance between the Intervention and the Control groups in this trial. However, considering the PPS [19] score of 70 as the cut-off between mild and significant disease, we are then able to assume that both groups have a similar distribution regarding this subject, as 18 patients in the Intervention group (62.1%) and 20 patients in the Control group (68.9%) had a PPS score below 70. Therefore, we believe that the groups remain comparable, and the trial’s results are not affected by the patients’ clinical status.

All the participants knew the facultative character of the trial. However, some dyads of patient/caregiver possibly accepted and consented to their inclusion in the trial mainly as an act of kindness to the palliative team, than for the true interest to participate. The serious life events these families were passing through frequently overlapped other possible interests; therefore, it is possible that some of the patients’ or caregivers’ data in this trial reflect automatically written answers, ridden by intuition. The lack of reflection on the real meaning of the questions might decrease the results’ accuracy and restrict the inference of the results to the palliative care population. We hypothesize that all participants equally chose the 3 Scenarios in Sect. 1, due to this fact and their low health literacy levels. As the 3 scenarios represented irreversible medical conditions, it is possible that the participants were unable to make significant distinctions between them and therefore, equally chose the three.

We hypothesize that a larger sample might reach significant statistical results. Involving other palliative centers to reach a bigger sample, might achieve more precise estimates and allow the inference of the results to the general population.

## Conclusions

End-of-life conversations are difficult, particularly when patients face a progressive, fatal disease, as they must confront and accept their clinical prognosis. The caregivers’ role as the patients’ surrogates should be perfectly understood so that decisions made on behalf of the patients are concordant with their end-of-life preferences, as an extension of their decision-making autonomy until the end of their lives.

Our trial showed that the physicians’ promotion of the Advance Directives’ [6] discussion between patients and their caregivers tends to improve concordance between the caregivers’ decisions as surrogates and the patients’ last wishes, in 66.6% of the answers, although without statistical significance, as a possible consequence of a small sample size. Moreover, in the Intervention group, the Proportion of Agreement did not decrease in any question (in phase 2), contrary to the Control group.

We conclude that this trial’s results support the intervention by physicians, when using the Advance Directives’ [6] documents as a communication tool between patients and caregivers to promote more capable and better prepared caregivers as health surrogates.

## Data Availability

The datasets used and/or analyzed during the current study are available from the corresponding author on reasonable request.
